# Automatic Facial Palsy Diagnosis as a Classification Problem Using Regional Information Extracted from a Photograph

**DOI:** 10.3390/diagnostics12071528

**Published:** 2022-06-23

**Authors:** Gemma S. Parra-Dominguez, Carlos H. Garcia-Capulin, Raul E. Sanchez-Yanez

**Affiliations:** Department of Electronics Engineering, Universidad de Guanajuato DICIS, Salamanca 36885, Mexico; gs.parradominguez@ugto.mx (G.S.P.-D.); sanchezy@ugto.mx (R.E.S.-Y.)

**Keywords:** clinical decision support systems, computerized assessment, facial palsy detection, facial paralysis diagnose, machine learning, medical diagnosis, medical screening, severity grading

## Abstract

The incapability to move the facial muscles is known as facial palsy, and it affects various abilities of the patient, for example, performing facial expressions. Recently, automatic approaches aiming to diagnose facial palsy using images and machine learning algorithms have emerged, focusing on providing an objective evaluation of the paralysis severity. This research proposes an approach to analyze and assess the lesion severity as a classification problem with three levels: healthy, slight, and strong palsy. The method explores the use of regional information, meaning that only certain areas of the face are of interest. Experiments carrying on multi-class classification tasks are performed using four different classifiers to validate a set of proposed hand-crafted features. After a set of experiments using this methodology on available image databases, great results are revealed (up to 95.61% of correct detection of palsy patients and 95.58% of correct assessment of the severity level). This perspective leads us to believe that the analysis of facial paralysis is possible with partial occlusions if face detection is accomplished and facial features are obtained adequately. The results also show that our methodology is suited to operate with other databases while attaining high performance, even though the image conditions are different and the participants do not perform equivalent facial expressions.

## 1. Introduction

The inability to move the facial muscles on one or both sides is known as facial palsy or facial paralysis. This inability can be generated from nerve damage due to trauma, congenital conditions, or diseases like stroke, brain tumor, or Bell’s palsy. Patients with facial palsy exhibit problems with speaking, blinking, swallowing saliva, eating, or communicating through natural facial expressions because of a noticeable drooping of facial capabilities. In general, the diagnosis by a clinician relies on a visual inspection of the patient’s facial symmetry. The examination of facial palsy requires specific medical training to produce a diagnosis, and it could vary from practitioner to practitioner. This is a reason why, in recent years, automatic approaches based on computer vision and artificial intelligence have been emerging to provide an objective evaluation of the paralysis.

The wide variety of methodologies working with facial palsy found in the literature can be divided according to their primary task. For example, if the intention is to perform a binary classification to discriminate between healthy or unhealthy subjects (i.e., to detect facial palsy), or to perform a multi-class classification (i.e., to evaluate the level of paralysis). Automatic approaches based on computer vision and artificial intelligence that seek to detect facial palsy include the works in [1,2,3,4,5,6]. Binary classification can be performed with an objective other than detecting facial paralysis; for example, identifying the type of facial palsy that a patient suffers, as Barbosa et al. did in [2] when seeking to discriminate between peripheral palsy or central palsy.

Automatic approaches aiming to assess the severity of facial palsy require a scale to measure the nerve damage. Those well-known scales are the House-Brackmann (HB), Sunnybrook, Yanagihara, FNGS 2.0, and eFACE. In detail, the grading scales split the facial nerve damage into a series of discrete levels according to some strict measures. Those measures are related to the symmetry of the face while displaying a neutral expression or showing a set of voluntary facial muscle movements; they are also associated with secondary features such as synkinesis [7]. Some authors, to start testing their algorithms, split the level of facial palsy into fewer degrees, for example: healthy, low, and high degrees of paralysis; and later report their findings [8,9,10].

Usually, automatic approaches are designed through handcrafted features and classifiers. Using a similar method, this research focuses on features computed from facial landmarks. As facial landmarks, we refer to the key points extracted using facial models on a face previously detected on an image. Many facial models (often referred to as shape predictors) are publicly available and can locate facial landmarks. Matthews and Baker introduced a 68-point facial model in [11], which is widely known and employed in the facial analysis field. However, few implementations fail in predicting those key points from persons who have facial palsy because the model was trained using imagery from healthy persons [12,13,14]. Recently, some authors have been working on improving the available shape predictors to extract facial landmarks in palsy patients accurately. Particularly relevant to our research is the model developed and released by Guarin et al. [15], which was trained using imagery of palsy patients.

The methodologies in [2,13,14,16] are based on handcrafted features extracted from facial landmarks and the use of a wide variety of classifiers. The asymmetry of the face is a core trait in those methodologies to diagnose facial palsy. Additionally, we wish to emphasize that the detection of facial paralysis was performed on proprietary databases with patients suffering from diverse levels of paralysis. To our knowledge those databases are unavailable to the research community, thus, a direct comparison of the performance is not possible. Some methodologies operate on specific facial gestures [13] and others require a set of movements to output a result [2,14,16]. Our proposed methodology is also based on handcrafted features, and they do not depend on a specific facial gesture to perform a classification task.

Diverse neural network structures have been applied to solve a variety of problems in the medical field [6,17,18,19,20,21,22,23,24]. Particularly, there are a few methodologies working with deep neural networks to evaluate facial palsy (e.g., [8,10,25,26,27,28]). Deep learning methods can improve the facial palsy detection rate, but their efficiency is limited by insufficient data and class imbalance, according to [28]. These methods automatically learn discriminative features, meaning that they do not compute handcrafted features but perform some pre-processing steps before training and evaluating the network. Due to the enormous amount of required data, geometric and color transformations, cropping, and resizing of the images are needed to increase the number of samples.

The methodologies in [10,26,28] are evaluated using the same facial palsy database; however, their experimental settings drastically differ in the testing method: leave-one-out protocol in [26], five-fold cross-validation in [10], and ten times repetitions in [28]. With this in mind, circumspect evaluations must be made when comparing performance. Both Hsu et al. [26] and Abayomi-Alli et al. [28] based their approach on well-known pre-trained networks with thousands of images. Particularly, Abayomi-Alli et al. extract 1000 deep features from a network’s final layer, and those are fed to a classifier. Our proposed methodology requires a simpler classifier structure with few features and samples to perform a classification task.

The analysis of the human face in terms of symmetry/asymmetry is not a new topic, and there is plenty of literature about it [29,30]. We talk about the symmetry of a person’s face knowing that we all are asymmetric. In other words, facial asymmetry responds to the fact that the left and right sides of our face showing no movement or while performing an expression are not identical. This asymmetry can be the result of various factors, including anatomical, neurological, physiological, pathological, psychological, and socio-cultural variables [29]. Usually, the growth and development of bones, nerves, and muscles should not produce new asymmetries.

We assume in this research that healthy faces have quite an identical left and right sides with a neutral expression, while the affected ones do not, allowing us to characterize the face of a palsy patient. In other words, a threshold value can be obtained using machine learning algorithms to determine to what extent the facial asymmetry is expected and where it is considered an unhealthy condition.

Our previous work in [6] introduced a system to detect facial palsy using 29 handcrafted geometrical features and a classifier based on neural networks. The objective was to classify face images into healthy and unhealthy regarding the severity of facial paralysis. There, the methodology operates on the assumption that facial paralysis can be characterized by locating levels of asymmetry in the face; if found, then it is said that the subject suffers from paralysis. The algorithm was evaluated in two different databases, obtaining a performance of 94.06% correct classification for the first database and 97.22% on the second one.

In this research, we also take the following perspectives in analyzing facial palsy. Some methodologies work with specific regions of the face (e.g., the mouth, the eyes, the forehead), and others extract helpful information from the entire face. Some approaches require a set of specific gestures to operate, and few need only one image to output a result. Finally, some methodologies perform binary discrimination between healthy and unhealthy subjects, and others evaluate different levels of paralysis based on a predefined clinical scale.

In this paper, we show that the framework introduced in [6] is independent of the facial gesture displayed by the person and that only one image is sufficient to output a result. We also show that our approach can perform multi-class classification tasks, and that the assessment of facial paralysis is possible with partial occlusions of the face if the analysis is executed on certain regions of the face. A performance analysis using four different classifiers is elaborated attaining excellent results to corroborate our features’ validity in a wide range of situations.

The main contributions of this work are: (1) a methodology to design classifiers based on easy-to-compute features that do not require a set of facial gestures to output a result, (2) a framework to analyze facial palsy using information extracted from the entire face or from specific facial regions (the eyes or the mouth), (3) a performance analysis using four different classifiers with excellent results which could provide orientation in selecting the best classification approach, and (4) evaluations on publicly available databases.

The rest of this paper is organized as follows. Section 2 introduces the proposed methodology. Section 3 describes the databases employed in this research and introduces the experiments, findings, and discussion. Finally, concluding remarks are provided in Section 4.

## 2. Methodology

**Figure 1:**
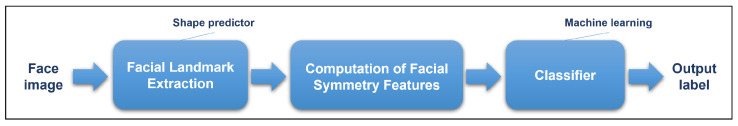
Framework of the proposed facial palsy assessment system.

The framework of the facial palsy assessment system under consideration is depicted in Figure 1. The methodology starts with face detection within the input image, followed by the extraction of the facial landmarks using a shape predictor. The proposed 29 facial symmetry features are subsequently computed using these key points. Such features are fed to four different classifiers, trained depending on the system’s goal. Detailed information will be provided next.

### 2.1. Facial Landmark Extraction

The first step of the module is to detect the face within the image, which is achieved using the default face detector implemented in the open-source *dlib C++ Library*. That particular face detector was designed through a combination of a linear classifier with the Histogram of Oriented Gradients descriptor [31], an image pyramid, and a sliding-window detection scheme; further information can be found at [32]. The second step extracts the facial landmarks using the shape predictor proposed by Guarin et al. and introduced in [15]. The complete extraction process is as follows:

1. Transform the input image to gray levels.
2. (Optional) Resize the transformed image according to a scale factor (sf) of $sf=\frac{W}{nW}$, having nW=200 and $nH=\frac{H}{sf}$. Where *W* and *H* refer to the width and height of the input image.
3. Detect the face rectangle on the smaller image.
4. (Optional) Re-scale the detected face rectangle to its original size using sf.
5. Extract the facial landmarks on the transformed image.
6. Store the predicted information for future processing.

**Figure 2:**
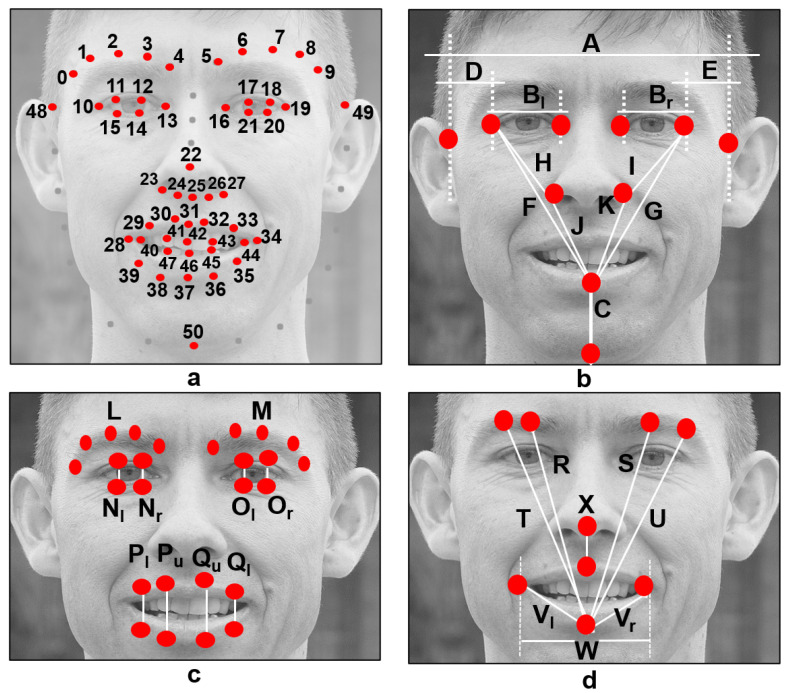
(**a**) The 51 key points inspired by the model proposed by Matthews and Baker [11]; (**b**–**d**) Facial distances to obtain spatial relations between facial landmarks [6].

Note that the shape predictor is a 68-point model, but only 51 points are of interest in this work. As sketched in Figure 2a, the 51 points are renumbered to ease the further calculation of attributes.

It is known that the head’s tilt angle can influence the accuracy of the facial symmetry quantification [16]. Thus, a tilt correction is performed using two known points and a transformation matrix before computing any symmetry measure. The left corner of the left eye and the right corner of the right eye (points 10 and 19 as seen in Figure 2a) are set as known points. The complete process to correct the head’s tilt angle is as follows:

1. Set as input data the eye-corner points.
2. Set the destination data on such points.
3. Calculate a transformation matrix $T_{f}$ using the eye-corner points and the similarity transform approach.
4. (Optional) Transform the input image using the $T_{f}$ matrix.
5. Rotate the predicted landmarks using the transformation $T_{f}$ matrix.

The landmark rotation process can be performed following the multiplication of matrices stated in Equation (1)

(1) $$\left(\begin{array}{c} Pir_{x}\\ Pir_{y} \end{array}\right)=\left[\begin{array}{ccc} T_{f(1,1)} & T_{f(1,2)} & T_{f(1,3)}\\ T_{f(2,1)} & T_{f(2,2)} & T_{f(2,3)} \end{array}\right]\left[\begin{array}{c} Pi_{x}\\ Pi_{y}\\ 1 \end{array}\right]$$

### 2.2. Computation of Facial Symmetry Features

Computing and analyzing the asymmetry found within both sides of the face, left and right, has shown to be helpful when aiming to detect palsy regions; also, when seeking to evaluate the patient lesion’s severity. The methodology mainly compares and quantifies the differences between both sides, specifically, the location and position of the facial organs (i.e., eyebrows, eyes, nose, and mouth). Initial tests led us to conclude that the regions of the eyebrows, eyes, and mouth provide meaningful information for this challenge, as reported by others [2,10,16,26]. Notice that some approaches require a set of images from the same subject performing specific gestures to operate (e.g., [2,4]), although it is strongly believed that the facial palsy assessment should not be conditioned to specific facial movements. Similar to [8], when a face image is loaded into our system, the facial gesture performed by the person does not need to be identified; therefore, the output of the system is a label obtained after an objective evaluation.

In total, 28 distances and two average values are calculated using the predicted key points, as depicted in Figure 2b–d. Distances A to K are influenced by the research of Ostrofsky et al. [33], who focused on evaluating objective measures from face photographs with an intention other than facial paralysis detection, but they seem to be an excellent hint to represent the healthy human face. The rest of the distances (L to W), in Figure 2c,d, were found helpful in our previous work [6] to specifically provide independence from the facial movement executed by the subject.

In this research, we assume that a healthy face is pretty symmetric concerning the shape and position of the face elements, independently of the subject’s facial gesture. If those elements are not symmetric, it is presumed that a grade of paralysis will be detected. Locating the affected side of the face is beyond the scope of this research.

The 29 proposed symmetry features are extracted using the 28 distances and the two average values introduced in Table 1. If additional information is required, please refer to Figure 2. Most of the computed distances provide ratios in the [0,1] range, here 0 means somewhat asymmetric, and 1 means closer to a healthy face. Analogous ratios between the left and right sides are later compared, and the maximum value is selected; these are the features described as “Max” in Table 1. Other features were designed to represent the inclination between two key points, particularly in the eyebrows; these are the features described as “slope”. It is expected for a healthy face to have a slope close to 0. Similarly, a few angles are computed between two key points. Again, it is expected for a healthy face to show an angle close to 0 (to be in a horizontal position), except for feature f23, which is expected to be vertical on a healthy subject. The features described as “ratio” reflect the asymmetry of the face; smaller values relate to a healthy subject and bigger values to an unhealthy one.

The facial symmetry features are computed according to Equation (2) for the angle between key points, Equation (3) for the slope of key points, the Euclidean distance is used here and calculated according to Equation (4), and the perimeter of a closed shape is computed using Equation (5):

(2) ∠(Pa,Pb)=arctan2(▵x,▵y)×180/π

where $▵x=Pa_{x}-Pb_{x}$ and $▵y=Pa_{y}-Pb_{y}$.

(3) $$m\left(Pa,Pb\right)=\left|\frac{Pa_{y}-Pb_{y}}{Pa_{x}-Pb_{x}}\right|$$

(4) $$d\left(Pa,Pb\right)=\sqrt{{\left(Pa_{x}-Pb_{x}\right)}^{2}+{\left(Pa_{y}-Pb_{y}\right)}^{2}}$$

(5) $$\bar{S}\left(P_{s},\ldots ,P_{e}\right)=\sum_{x=s}^{l-1}d\left(P_{x},P_{x}+1\right)+d\left(P_{s},P_{e}\right)$$

where $\bar{S}$ is a closed shape, $P_{s}$ is the start point, and $P_{e}$ is the endpoint within the shape.

**Figure 3:**
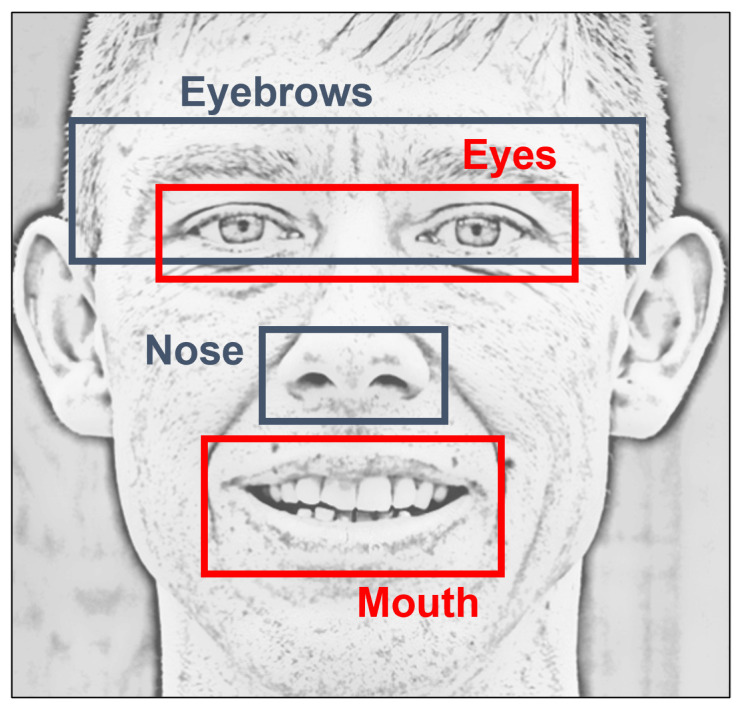
Example of a face image divided into four facial regions.

The use of regional information is explored in this paper to detect and evaluate facial paralysis. As mentioned before, a number of approaches extract meaningful information from the entire face; but it is also possible to extract useful information from specific areas of the face. Some of those areas are the eyebrows, the eyes, the nose, and the mouth, as shown in Figure 3. We refer to the features computed from those facial areas as regional information. Particularly in this research, experiments are carried out on the entire face, the eyes, and the mouth. Here, it is referred to as the entire face to the use of the 29 proposed symmetry features to execute classification tasks. For the eyes, only 19 features are considered and they correspond to features named as Eyebrows, Eyes, Nose, and Combined (only *f23* and *f25*–*f27*) in Table 1. Consequently, the 15 features that correspond to the mouth are all features named Mouth, Nose, and Combined in Table 1.

### 2.3. Classification

Our proposed classifiers were designed using the Waikato Environment for Knowledge Analysis (Weka) suite. Weka consists of a collection of machine learning algorithms for data mining tasks. It includes tools for data preparation, classification, regression, clustering, association rules mining, and visualization. In this work, four classifiers were configured based on the multi-layer perceptron (MLP), support vector machine (SVM), k-nearest-neighbor (KNN), and multinomial logistic regression (MNLR) methods. Depending on the implementation, each classifier requires specific parameters to operate, and those are optimized to reach the best performance. A list of some of those parameters is described in Table 2. More information concerning the Weka suite can be found at [34].

The Weka suite operates using Attribute-Relation File Format (arff) files, which are text files that describe a list of samples sharing a set of features and labels, for more information about how to create them, refer to [35]. In this work, after computing the data set composed of features extracted from healthy and palsy patients, an arff file for each training and testing set was created by running an easy-to-implement script. Then the files are loaded into Weka, and the training process begins, ending with the evaluation process. Further details on the tests and results are provided next.

## 3. Results and Discussion

A few methodologies in the literature look to assess facial paralysis in an image. Then, it seems relevant to mention that collaborating with the research community has been complicated due to the unavailability of public databases, mainly because of the need of preserving the patient’s privacy. This scenario encouraged us to evaluate our system on a database that is publicly available. As stated previously, this research seeks to expand our previous methodology to perform the tasks of detection and assessment of facial palsy regions. In this work, three experiments were executed to evaluate the performance of four different classification methods. The results and findings are discussed in the following paragraphs.

Comments on the database where images of palsy patients were extracted are given now. The YouTube Facial Palsy (YFP) database is an image collection provided by Hsu et al. [9]. The YFP database is a compilation of 32 videos from 21 patients obtained from YouTube; 10 additional patients in a second release were included. The patient talks to the camera in each video, and the facial expression variation across time is recorded. Each video was converted into an image sequence at 6 FPS, yielding almost 3000 images. Three independent clinicians labeled the palsy regions in each frame; the junction of the independently cropped areas is considered the ground truth. The authors also provided additional labels to classify the intensity exhibited in each palsy region. It is our understanding that the authors in [9] determined these intensity labels; they did not declare that the intensity label was approved by a clinician, which could lead to discrepancies in the classification results among methodologies. The YFP is available upon request at [36].

The Extended Cohn-Kanade (CK+) database distribution, a well-known database in the research community to prototype and benchmark systems for the automated detection of facial expression [37], was also employed. The CK+ database collects 593 sequences across 123 subjects, close to 10,800 images, and all sequences go from a neutral face to a peak expression. The CK+ database is included in this work, with the YFP database, to make our methodology robust against expression variation as suggested in [9,10]. The unhealthy samples came from the YFP database and the healthy subjects from the CK+.

Widely known evaluation metrics are computed to measure the performance of each classifier. These are accuracy, recall (or true positive rate), precision, F1 score, true negative rate, false negative rate, and false positive rate which are calculated according to Equations (6)–(12), respectively.

(6) $$Acc=\frac{TP+TN}{TP+TN+FP+FN}$$

(7) $$Rec=\frac{TP}{TP+FN}$$

(8) $$Prec=\frac{TP}{TP+FP}$$

(9) $$F1s=\frac{2\times TP}{2\times TP+FP+FN}$$

(10) $$TNR=\frac{TN}{TN+FP}$$

(11) $$FNR=\frac{FN}{FN+TP}$$

(12) $$FPR=\frac{FP}{FP+TN}$$

where *TP* is short for true positives, *TN* for true negatives, *FP* for false positives, and *FN* for false negatives.

The 5-fold cross-validation protocol was adopted to test the model accuracy for each classification task. Such protocol allows us to test on unseen samples, reducing the possibility of over-fitting to previously seen ones. This cross-validation strategy splits the data set into five subsets. Every subset is preserved as validation data, and the other four are used as training data, ensuring that the test data is untouched in each experiment occurrence. The experiment is repeated five times, where each subset has the same probability for validation. The accumulation of correct classified samples measures the performance.

### 3.1. Palsy Region Detection

The first experiments are focused on detecting facial palsy, in other words, on classifying the input data as healthy or unhealthy. It is called palsy region detection because those particular algorithms analyze specific facial regions. Here, the experiments evaluate our methodology using regional information, but our initial proposal is to inspect the whole face using our symmetry features. Three tests were performed: (1) the detection of palsy using 29 features, (2) the detection of palsy in the eyes, and (3) the detection of palsy in the mouth. As described earlier, the regional information for the eyes correspond to features named as Eyebrows, Eyes, Nose, and Combined (only *f23* and *f25*–*f27*) in Table 1. Similarly, the regional information for the mouth corresponds to all features, except those named as Eyebrows and Eyes features in Table 1.

In general, the data set is composed of 19 palsy patients and 19 healthy subjects. The palsy patients are subjects 1, 5, 6, 7, 11, 12, 13, 14, 15, 19, 20, 21, 23, 24, 25, 28, 29, 30, and 31 from the YFP database. Patients with less than 20 images and patients with facial occlusions were excluded. The healthy subjects belong to the S022, S026, S028, S034, S042, S046, S050, S054, S057, S102, S105, S124, S130, S131, S132, S133, S134, S135, and S136 folders in the CK+ collection.

The data set for the first experiment comprises 20 images from each of the 38 participants (760 images in total); this arrangement is expected to have the same amount of healthy and palsy samples, making it a balanced data set for the experiment. Notice that the healthy subjects are labeled as class 0 and the palsy subjects as class 1. For this experiment, the classifiers were configured as described in Table 3. The experiment using 5-fold cross-validation was repeated 10 times, and the average performance is shown in Table 4, for the three tests.

Great results are obtained for the MLP and SVM classifiers, 95.03% and 95.61%, respectively. Few samples were required during the training phase compared to other approaches that required thousands of images to output a label. Good results are also reached using only regional features, in the eye and the mouth, 93.42% and 92.98%, respectively. Still, the entire face analysis is better than focusing on a single region when discriminating between healthy and unhealthy subjects.

**Figure 4:**
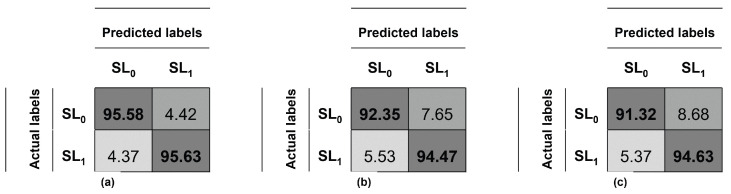
Confusion matrix of the detection of the palsy: (**a**) on the entire face, (**b**) on the eyes region and (**c**) on the mouth region.

The confusion matrix of this experiment using the entire face and SVM is depicted in Figure 4a, and the system’s average accuracy is 95.61%, recall 95.63%, precision 95.61% and F1-score 95.62%. The confusion matrix analyzing the eyes with SVM is depicted in Figure 4b, and the system’s average accuracy is 93.42%, recall 94.47%, precision 92.55% and F1-score 93.50%. Finally, the confusion matrix analyzing the eyes with MLP is depicted in Figure 4c, and the system’s average accuracy is 92.98%, recall 94.63%, precision 91.64% and F1-score 93.11%.

Additional performance results for the experiments are provided on Table 5. For our methodology, the true negative rate (TNR) reflects the number of healthy subjects detected as normal participants; while the true positive rate (TPR) shows the number of palsy patients detected as unhealthy subjects. Great results are achieved using the SVM classifier: 95.59% and 95.63% for the face, 92.35% and 94.47% for the eyes. On the other hand, the false negative and false positive rates are expected to be as lower as possible because both of them represent a wrong diagnosis. Good results are obtained for this metric: 4.37% and 4.41% for the face, 5.53% and 7.65% for the eyes. Similarly, good results are reached for the mouth using the MLP classifier, 91.32% and 94.63% of true detection rates; and 5.37% and 8.68% of false detection rates.

Although out of the scope of this work, those scores using either the eye or mouth information lead us to believe that we can use these features to detect facial palsy on images with partial occlusions. If face detection is achieved and landmarks are predicted adequately, an analysis to detect facial palsy might be possible. In other words, our analysis allows us to determine to what extent regional information is needed to diagnose the severity of the lesion with satisfactory results.

### 3.2. Prediction of Two Palsy Levels

The second experiment seeks to distinguish between two levels of facial palsy. As stated by Hsu et al. [9], these levels are slight (or low-intensity) and strong (or high-intensity) facial palsy. The authors provided labels for the mouth and the eyes regions; there might be a case where the intensity is not the same for both regions, then a separate analysis is required. The data set is composed of 19 patients from the YFP database and is now divided into class $SL_{1}$ (low-intensity) and class $SL_{2}$ (high-intensity). After preliminary tests, it was found that 40 images per patient were adequate to train the classifier, but for those who did not have enough images, only 20 were employed. To improve the learning process, a data augmentation was performed as suggested in [1,16]. This process consisted of rotating in two opposite directions the palsy images to increase the amount of available data. This augmentation also allows us to verify that the algorithm is invariant to rotation, as stated in Section 2.1. This experiment is divided into two tests (1) using the 29 proposed features and (2) using regional information (19 features for the eyes and 15 features for the mouth).

The data distribution for this experiment is described in Table 6. There, to evaluate the level of paralysis in the eyes, 208 low-intensity and 472 high-intensity images are included. After data augmentation, the data set is formed by 624 low-intensity and 1416 high-intensity samples (2040 images in total). Similarly, to evaluate the level of paralysis in the mouth, 141 low-intensity and 539 high-intensity images are included. After data augmentation, the data set is formed by 423 low-intensity and 1617 high-intensity samples (2040 images in total).

For the first test, the configuration of the classifiers is described in Table 7. A 5-fold cross-validation was performed and repeated 10 times, and the average performance is shown in Table 8 for both regions. Great results are obtained assessing the eyes region, up to 95.05% using SVM. Similarly, good results are reached for the mouth area, 92.69%. In this task, the KNN classifier reached better results than the MLP for both cases. Still, the proposed MLP yielded good results using few samples, compared to other published deep learning approaches that require thousands of images and complex neural network structures.

For the second test, the configuration of the classifiers is described in Table 9. Again a 5-fold cross-validation repeated 10 times was performed, and the average performance is shown in Table 10 for both regions using fewer features. It was expected a lower performance because the information feed to the classifiers was decreased; still, good performance (more than 90%) was achieved using SVM. A slight increase in performance is observed when using the information from the mouth region. This evaluation leads us to believe that a classification of the palsy intensity is possible to a certain degree, in partial occlusions of the face.

### 3.3. Prediction of Three Palsy Levels

**Figure 5:**
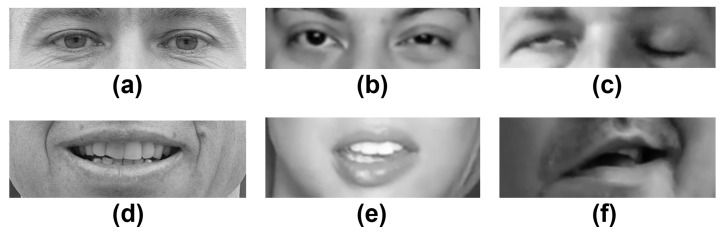
Facial analysis: (**a**) healthy eyes, (**b**) slight palsy and (**c**) strong palsy eyes; (**d**) healthy mouth, (**e**) slight palsy, and (**f**) strong palsy mouth. Palsy images were obtained from [9].

The goal of assessing the severity of the lesion is to determine how diminished the facial nerve function is. It can be evaluated once the palsy has been detected or evaluated at the same time. For the third experiment, the intensity labels provided by Hsu et al. [9] were also used. As previously mentioned, the authors offer labels for the mouth and the eyes’ regions independently, and there might be a case where the intensity label is not the same for both regions. The data set is now divided into class $SL_{0}$ (healthy), class $SL_{1}$ (slight palsy or low-intensity), and class $SL_{2}$ (strong palsy or high-intensity). In Figure 5, a sample of healthy subjects and patients who have facial palsy is introduced. Specifically, the images show the two facial palsy regions that are of interest in this work: the eyes and mouth.

This experiment is also divided into two tests (1) using the 29 proposed features and (2) using regional information (19 features for the eyes and 15 features for the mouth). The data set is composed of 19 patients from the YFP database and 19 participants from the CK+ database. In this case, 40 images per subject were used; for those who did not have enough images, only 20 were employed. Once more, the same data augmentation process was performed to provide enough information to the classifiers, increasing the amount of data and verifying that the methodology is rotation invariant.

The data distribution is described in Table 11. For the eyes’ region, 740 healthy, 208 low-intensity, and 472 high-intensity samples are included; it is easy to observe that the classes are unbalanced and that there are few examples for the class $SL_{1}$. After augmenting the samples, 4260 images compose the training set with 2220 healthy, 624 low-intensity, and 1416 high-intensity samples. For the mouth region, 740 healthy samples, 141 low-intensity, and 539 high-intensity samples are included; again, there are fewer examples for the class $SL_{1}$. After augmenting the samples, 4260 images compose the training set with 2220 healthy, 423 low-intensity, and 1617 high-intensity samples. In both tests, the classes remained unbalanced, but after several evaluations, this data distribution provided the best results.

The configuration of the classifiers is described in Table 12. The experiment using 5-fold cross-validation was repeated 10 times, and the average performance is shown in Table 13, for both regions. Great results are obtained assessing the eyes region, up to 95.58% using SVM. Similarly, good results are reached for the mouth area, 94.44%. In both cases, the proposed MLP yielded similar results using few samples, compared to other deep learning strategies that require thousands of samples and complex neural network structures.

For the second test, the configuration of the classifiers is described in Table 14. Again a 5-fold cross-validation was performed and repeated 10 times, and the average performance is shown in Table 15 for both regions using fewer features. It was expected a lower performance because the information fed to the classifiers was decreased; still, good performance, 92.08% and 93.95% were achieved using SVM. Once more, this evaluation leads us to believe that a classification of the palsy intensity is possible to a certain degree, in partial occlusions of the face.

## 4. Conclusions

A methodology to assess facial paralysis in an image was proposed. It is assumed that facial palsy can be interpreted as a problem of asymmetry levels between the elements of the face, particularly the eyebrows, eyes, and mouth. The proposed assessment system consists of 29 facial symmetry features extracted from predicted landmarks and a classifier that provides a label as an output. Four different classifiers were evaluated in three experiments to validate our methodology. Those experiments seek to detect facial palsy, discriminate among two levels of the palsy, and assess the lesion’s severity among three levels of palsy. The best results were achieved using SVM, but a similar performance with a slight decrease is obtained using the multi-layer perceptron approach. After the evaluations, it was found that dividing the face into specific regions is convenient to detect and assess the paralysis with fewer features. This feature reduction leads us to believe that the analysis of facial paralysis is possible with partial occlusions of the face, as long as face detection is achieved, and landmarks are predicted adequately.

To validate the proposed methodology, tests were performed on publicly available image databases, the YouTube Facial Palsy (YFP) with 21 participants with facial palsy, and the CK+ with 123 healthy subjects. In the first classification task, binary discrimination between healthy and unhealthy subjects, the proposed system achieved the highest accuracy of 95.61% after evaluating using the 5-fold cross-validation protocol. In a second task, a binary classification to detect the intensity of the facial palsy (low-intensity vs. high-intensity), the system achieved the highest accuracy of 95.05% in the eyes and 93.29% in the mouth region. Finally, in a third task to classify the severity of the damage (healthy, low-intensity, and high-intensity), the system achieved the highest accuracy of 95.58% for the eyes and 94.44% for the mouth.

It has been noted that evaluating facial paralysis using symmetry/asymmetry values is risky because the human face is not identical concerning its left and right sides. Then, to thoroughly verify the usefulness of our algorithm in clinical practice, a much larger sample of healthy controls with different degrees of facial asymmetry (not caused by facial palsy) is needed. Achieving this monumental task would require a specific database of healthy participants (i.e., showing no facial palsy of any kind) and a multidisciplinary team of experts to design a grading scale of healthy asymmetry, to calculate how asymmetric is the subject’s face according to it, and to label each participant’s image manually. To the best of our knowledge, no such data set is available for the research community.

Future work would include additional evaluations on available databases, with annotated data, of palsy patients and healthy controls. The design of a mobile application to diagnose facial palsy at home is also desirable to improve the rate of early diagnosis. Furthermore, developing an application to easily monitor the treatment and improvement of the patient would represent a milestone.

To conclude, the accomplished results show that the proposed methodology to design classifiers can be adapted to other data sets with outstanding results. It is a methodology that is easy to replicate compared to the other complex systems and achieves similar results for detecting and evaluating facial paralysis. Finally, the proposed classifiers require fewer samples in the training stage compared to different approaches based on deep neural networks. The code to compute the 29 facial symmetry features and the trained models are available upon request; notice that the image databases must be requested from the rightful owners at [36,37].

## Figures and Tables

**Table 1:** Facial symmetry features, introduced by Parra-Dominguez et al. [6].

No.	Facial Region	Type	Formula
*f0*	Eyebrows	Angle	|∠(P0,P9)|
*f1*	Eyebrows	Angle	|∠(P2,P7)|
*f2*	Eyebrows	Angle	|∠(P4,P5)|
*f3*	Eyebrows	Max.	max(L/M,M/L)
*f4*	Eyebrows	Slope	m(P0,P9)
*f5*	Eyebrows	Slope	m(P2,P7)
*f6*	Eyebrows	Slope	m(P4,P5)
*f7*	Eyes	Angle	|∠(P10,P19)|
*f8*	Eyes	Max.	$\max(B_{l}/B_{r},B_{r}/B_{l})$
*f9*	Eyes	Max.	max(D/E,E/D)
*f10*	Eyes	Max.	max(H/I,I/H)
*f11*	Eyes	Max.	max(N/O,O/N)
*f12*	Eyes	Max.	$\max(N_{l}/O_{r},O_{r}/N_{l})$
*f13*	Eyes	Max.	$\max(N_{r}/O_{l},O_{l}/N_{r})$
*f14*	Mouth	Angle	|∠(P28,P34)|
*f15*	Mouth	Max.	max(F/G,G/F)
*f16*	Mouth	Max.	$\max(P_{l}/Q_{l},Q_{l}/P_{l})$
*f17*	Mouth	Max.	$\max(P_{u}/Q_{u},Q_{u}/P_{u})$
*f18*	Mouth	Max.	$\max(V_{l}/A,V_{r}/A)$
*f19*	Mouth	Max.	$\max(P_{l}/W,Q_{l}/W)$
*f20*	Mouth	Max.	$\max(P_{u}/W,Q_{u}/W)$
*f21*	Mouth	Max.	$\max(W_{l}/W,W_{r}/W)$
*f22*	Nose	Angle	|∠(P23,P27)|
*f23*	Combined	Angle	|∠(P22,P37)|
*f24*	Combined	Max.	max(J/K,K/J)
*f25*	Combined	Max.	max(T/A,U/A)
*f26*	Combined	Max.	max(R/A,S/A)
*f27*	Combined	Ratio	C/A
*f28*	Combined	Ratio	X/A

In *f3*, *L* and *M* are the average height of all the left and right eyebrow points, respectively. In *f11*, *N* = (*N_l_* + *N_r_*)/2, similarly, *O* = (*O_l_* + *O_r_*)/2. In *f19*, *f20*, and *f21*, *W* is the distance shown in Figure 2d, and the perimeter values *W_l_* and *W_r_* are computed as *W_l_* = $\bar{S}$(*P28*, *P29*, *P30*, *P31*, *P37*, *P38*, *P39*) and *W_r_* = $\bar{S}$(*P31*, *P32*, *P33*, *P34*, *P35*, *P36*, *P37*).

**Table 2:** Required parameters to operate the classifier in the Weka suite, according to [34].

Method	Parameters	Weka Function
MLP	Learning rate (L), momentum (M), training time (N), number of neurons in the hidden layers (H), and seed (S)	MultilayerPerceptron
SVM	Cost (C), gamma (G), kernel type	LibSVM
KNN	Number of neighbors (KNN) and distance function (A)	IBk
MNLR	Ridge (R)	Logistic

In Weka, the parameter N of MLP refers to the number of epochs to train through and the nodes in the network are all sigmoid. Additionally, the radial basis function (RBF) kernel was used in all experiments using SVM and only one neighbor was set for the KNN classifier.

**Table 3:** First experiment: classifiers’ configuration.

Method	Parameters
MLP	L=0.2045, M=0.1909, H=59, N=5000, S=0
SVM	C=1000, G={0.1,0.001,0.01} *, kernel type = RBF
KNN	KNN =1, A= Euclidean distance
MNLR	$R=1\;\times \;10^{-8}$

* Refers to the gamma value for the face, eyes, and mouth evaluation, respectively.

**Table 4:** Results of the detection of palsy regions on the criteria of accuracy.

Classifier	Face	Eyes	Mouth
MLP	95.03±1.69%	92.86±1.99%	92.98±2.13%
SVM	95.61±1.40%	93.42±1.84%	90.93±2.49%
KNN	92.34±1.80%	89.33±2.24%	91.28±2.21%
MNLR	94.24±1.60%	92.16±1.99%	91.07±2.35%

**Table 5:** Performance results for the detection of palsy regions.

Region	Classifier	TNR	FNR	TPR	FPR
Face	SVM	95.59±2.28%	4.37±2.05%	95.63±2.05%	4.41±2.28%
Eyes	SVM	92.35±3.11%	5.53±2.81%	94.47±2.81%	7.65±3.11%
Mouth	MLP	91.32±3.51%	5.37±2.85%	94.63±2.85%	8.68±3.51%

**Table 6:** Data distribution for the prediction of two palsy levels.

Test	Total of Images	Data Distribution
Eyes region	680	Original data: 208 low-intensity and 472 high-intensity samples
	2040	Augmented data: 624 low-intensity and 1416 high-intensity samples
Mouth region	680	Original data: 141 low-intensity and 539 high-intensity samples
	2040	Augmented data: 423 low-intensity and 1617 high-intensity samples

**Table 7:** Second experiment: classifiers’ configuration for the first test.

Method	Parameters
MLP	L=0.2045, M=0.1909, H=59, N={500,2000} *, S={2,37} *
SVM	C=1000, G=1.0, kernel type = RBF
KNN	KNN =1, A= Manhattan distance
MNLR	$R=1\;\times \;10^{-8}$

* Refers to the values for the eyes and mouth evaluation, respectively.

**Table 8:** Results on the prediction of two palsy levels on the criteria of accuracy using the 29 symmetry features.

Classifier	Eyes	Mouth
MLP	92.39±1.23%	90.20±1.27%
SVM	95.05±1.14%	92.69±1.01%
KNN	93.54±1.24%	92.14±1.16%
MNLR	82.96±1.83%	81.09±1.47%

**Table 9:** Second experiment: classifiers’ configuration for the second test.

Method	Parameters
MLP	L=0.2045, M=0.1909, H=59, N={1000,500} *, S={87,7} *
SVM	C=10, G=1.0, kernel type = RBF
KNN	KNN =1, A= Manhattan distance
MNLR	$R=1\;\times \;10^{-8}$

* Refers to the values for the eyes and mouth evaluation, respectively.

**Table 10:** Results on the prediction of two palsy levels on the criteria of accuracy using regional information.

Classifier	Eyes	Mouth
MLP	88.75±1.87%	89.60±1.27%
SVM	91.12±1.57%	93.29±1.22%
KNN	89.99±1.54%	91.70±0.94%
MNLR	79.97±1.97%	80.15±1.13%

**Table 11:** Data distribution for the prediction of three palsy levels.

Test	Total of Images	Data Distribution
Eyes region	1420	Original data: 740 healthy, 208 low-intensity and 472 high-intensity samples
	4260	Augmented data: 2220 healthy, 624 low-intensity and 1416 high-intensity samples
Mouth region	1420	Original data: 740 healthy, 141 low-intensity and 539 high-intensity samples
	4260	Augmented data: 2220 healthy, 423 low-intensity and 1617 high-intensity samples

**Table 12:** Third experiment: classifiers’ configuration for the first test.

Method	Parameters
MLP	L=0.2045, M=0.1909, H=59, N={1000,7000} *, S={18,25} *
SVM	C={10,1000} *, G=1.0, kernel type = RBF
KNN	KNN =1, A= Manhattan distance
MNLR	$R=1\;\times \;10^{-8}$

* Refers to the values for the eyes and mouth evaluation, respectively.

**Table 13:** Results on the palsy lesion assessment on the criteria of accuracy using the 29 symmetry features.

Classifier	Eyes	Mouth
MLP	93.67±0.94%	92.77±0.87%
SVM	95.58±0.71%	94.44±0.63%
KNN	93.21±0.80%	92.94±0.80%
MNLR	86.48±0.82%	85.95±0.77%

**Table 14:** Third experiment: classifiers’ configuration for the second test.

Method	Parameters
MLP	L=0.2045, M=0.1909, H=59, N={4000,7000} *, S={18,25} *
SVM	C=10, G=1.0, kernel type = RBF
KNN	KNN =1, A={Manhattan, Euclidean distance}
MNLR	$R=1\;\times \;10^{-8}$

* Refers to the values for the eyes and mouth evaluation, respectively.

**Table 15:** Results on the palsy lesion assessment on the criteria of accuracy using regional information.

Classifier	Eyes	Mouth
MLP	89.63±1.04%	91.91±0.94%
SVM	92.08±0.79%	93.95±0.62%
KNN	89.24±0.92%	92.08±0.83%
MNLR	83.07±1.07%	84.30±0.76%

## Data Availability

Not applicable.

## References

[1] Kim H.S., Kim S.Y., Kim Y.H., Park K.S. (2015). A smartphone-based automatic diagnosis system for facial nerve palsy. Sensors.

[2] Barbosa J., Lee K., Lee S., Lodhi B., Cho J.G., Seo W.K., Kang J. (2016). Efficient quantitative assessment of facial paralysis using iris segmentation and active contour-based key points detection with hybrid classifier. BMC Med. Imaging.

[3] Song A., Xu G., Ding X., Song J., Xu G., Zhang W. (2017). Assessment for facial nerve paralysis based on facial asymmetry. Australas. Phys. Eng. Sci. Med..

[4] Barbosa J., Seo W.K., Kang J. (2019). paraFaceTest: An ensemble of regression tree-based facial features extraction for efficient facial paralysis classification. BMC Med. Imaging.

[5] Zhuang Y., McDonald M., Uribe O., Yin X., Parikh D., Southerland A.M., Rohde G.K. (2020). Facial Weakness Analysis and Quantification of Static Images. IEEE J. Biomed. Health Inform..

[6] Parra-Dominguez G.S., Sanchez-Yanez R.E., Garcia-Capulin C.H. (2021). Facial Paralysis Detection on Images Using Key Point Analysis. Appl. Sci..

[7] Thevenot J., López M.B., Hadid A. (2018). A survey on computer vision for assistive medical diagnosis from faces. IEEE J. Biomed. Health Inform..

[8] Song A., Wu Z., Ding X., Hu Q., Di X. (2018). Neurologist Standard Classification of Facial Nerve Paralysis with Deep Neural Networks. Future Internet.

[9] Hsu G.S.J., Kang J.H., Huang W.F. (2019). Deep Hierarchical Network With Line Segment Learning for Quantitative Analysis of Facial Palsy. IEEE Access.

[10] Liu X., Xia Y., Yu H., Dong J., Jian M., Pham T.D. (2020). Region Based Parallel Hierarchy Convolutional Neural Network for Automatic Facial Nerve Paralysis Evaluation. IEEE Trans. Neural Syst. Rehabil. Eng..

[11] Matthews I., Baker S. (2004). Active appearance models revisited. Int. J. Comput. Vis..

[12] Guarin D.L., Dusseldorp J., Hadlock T.A., Jowett N. (2018). A machine learning approach for automated facial measurements in facial palsy. JAMA Facial Plast. Surg..

[13] Jiang C., Wu J., Zhong W., Wei M., Tong J., Yu H., Wang L. (2020). Automatic facial paralysis assessment via computational image analysis. J. Healthc. Eng..

[14] Wang T., Zhang S., Yu H., Dong J., Liu L.A. (2016). Automatic evaluation of the degree of facial nerve paralysis. Multimed. Tools Appl..

[15] Guarin D.L., Yunusova Y., Taati B., Dusseldorp J.R., Mohan S., Tavares J., van Veen M.M., Fortier E., Hadlock T.A., Jowett N. (2020). Toward an automatic system for computer-aided assessment in facial palsy. Facial Plast. Surg. Aesthetic Med..

[16] Guo Z., Dan G., Xiang J., Wang J., Yang W., Ding H., Deussen O., Zhou Y. (2018). An unobtrusive computerized assessment framework for unilateral peripheral facial paralysis. IEEE J. Biomed. Health Inform..

[17] Azar A.T. (2013). Fast neural network learning algorithms for medical applications. Neural Comput. Appl..

[18] Kabir H.M.D., Khosravi A., Hosen M.A., Nahavandi S. (2018). Neural Network-Based Uncertainty Quantification: A Survey of Methodologies and Applications. IEEE Access.

[19] Albu A., Precup R.E., Teban T.A. (2019). Results and Challenges of Artificial Neural Networks used for Decision-Making and Control in Medical Applications. Mech. Eng..

[20] Smys S., Chen J.I.Z., Shakya S. (2020). Survey on Neural Network Architectures with Deep Learning. J. Soft Comput. Paradig. (JSCP).

[21] Izonin I., Tkachenko R., Gregus M., Ryvak L., Kulyk V., Chopyak V. (2021). Hybrid Classifier via PNN-based Dimensionality Reduction Approach for Biomedical Engineering Task. Procedia Comput. Sci..

[22] Parra-Dominguez G.S., Sanchez-Yanez R.E., Garcia-Capulin C.H. (2022). Towards Facial Gesture Recognition in Photographs of Patients with Facial Palsy. Healthcare.

[23] Izonin I., Tkachenko R., Duriagina Z., Shakhovska N., Kovtun V., Lotoshynska N. (2022). Smart Web Service of Ti-Based Alloy’s Quality Evaluation for Medical Implants Manufacturing. Appl. Sci..

[24] Taran V., Gordienko Y., Rokovyi O., Alienin O., Kochura Y., Stirenko S., Hu Z., Zhang Q., Petoukhov S., He M. (2022). Edge Intelligence for Medical Applications Under Field Conditions. Advances in Artificial Systems for Logistics Engineering.

[25] Guo Z., Shen M., Duan L., Zhou Y., Xiang J., Ding H., Chen S., Deussen O., Dan G. Deep assessment process: Objective assessment process for unilateral peripheral facial paralysis via deep convolutional neural network. Proceedings of the 2017 IEEE 14th International Symposium on Biomedical Imaging (ISBI 2017).

[26] Hsu G.S.J., Huang W.F., Kang J.H. Hierarchical network for facial palsy detection. Proceedings of the 2018 IEEE/CVF Conference on Computer Vision and Pattern Recognition Workshops (CVPRW).

[27] Sajid M., Shafique T., Baig M.J.A., Riaz I., Amin S., Manzoor S. (2018). Automatic grading of palsy using asymmetrical facial features: A study complemented by new solutions. Symmetry.

[28] Abayomi-Alli O.O., Dama`eviČius R., Maskeliūnas R., Misra S. (2021). Few-Shot Learning with a Novel Voronoi Tessellation-Based Image Augmentation Method for Facial Palsy Detection. Electronics.

[29] Borod J.C., van Gelder R.S. (1990). INTRODUCTION. Int. J. Psychol..

[30] Codari M., Pucciarelli V., Stangoni F., Zago M., Tarabbia F., Biglioli F., Sforza C. (2017). Facial thirds–based evaluation of facial asymmetry using stereophotogrammetric devices: Application to facial palsy subjects. J. Cranio-Maxillofac. Surg..

[31] Dalal N., Triggs B. Histograms of oriented gradients for human detection. Proceedings of the 2005 IEEE Computer Society Conference on Computer Vision and Pattern Recognition (CVPR’05).

[32] King D.E. (2009). Dlib-ml: A Machine Learning Toolkit. J. Mach. Learn. Res..

[33] Ostrofsky J., Cohen D.J., Kozbelt A. (2014). Objective versus subjective measures of face-drawing accuracy and their relations with perceptual constancies. Psychol. Aesthetics Creat. Arts.

[34] Witten I.H., Frank E., Hall M.A. (2011). Data Mining: Practical Machine Learning Tools and Techniques.

[35] Paynter G. (2002). Attribute-Relation File Format (ARFF). https://www.cs.waikato.ac.nz/ml/weka/arff.html.

[36] Hsu G.S. (2020). YouTube Facial Palsy (YFP) Database. https://sites.google.com/view/yfp-database/.

[37] Lucey P., Cohn J.F., Kanade T., Saragih J., Ambadar Z., Matthews I. The Extended Cohn-Kanade Dataset (CK+): A complete dataset for action unit and emotion-specified expression. Proceedings of the 2010 IEEE Computer Society Conference on Computer Vision and Pattern Recognition—Workshops.

